# XModNN: Explainable Modular Neural Network to Identify Clinical Parameters and Disease Biomarkers in Transcriptomic Datasets

**DOI:** 10.3390/biom14121501

**Published:** 2024-11-25

**Authors:** Jan Oldenburg, Jonas Wagner, Sascha Troschke-Meurer, Jessica Plietz, Lars Kaderali, Henry Völzke, Matthias Nauck, Georg Homuth, Uwe Völker, Stefan Simm

**Affiliations:** 1 Institute of Bioinformatics, University Medicine Greifswald, 17475 Greifswald, Germany; jan.oldenburg@uni-greifswald.de (J.O.); jonas.wagner@uni-greifswald.de (J.W.); lars.kaderali@uni-greifswald.de (L.K.); 2 Institute for Bioanalysis, Department of Applied Sciences, Coburg University of Applied Sciences and Arts, 96450 Coburg, Germany; 3 Institute for Pediatric Hematology and Oncology, University Medicine Greifswald, 17475 Greifswald, Germany; sascha.troschke-meurer@med.uni-greifswald.de (S.T.-M.); jessica.plietz@med.uni-greifswald.de (J.P.); 4 Institute for Community Medicine, University Medicine Greifswald, 17475 Greifswald, Germany; voelzke@uni-greifswald.de; 5 Institute of Clinical Chemistry and Laboratory Medicine, University Medicine Greifswald, 17475 Greifswald, Germany; matthias.nauck@med.uni-greifswald.de; 6 Interfaculty Institute for Genetics and Functional Genomics, University Medicine Greifswald, 17475 Greifswald, Germany; homuth@uni-greifswald.de (G.H.); voelker@uni-greifswald.de (U.V.)

**Keywords:** modular neural network, explainable AI, next-generation sequencing, biomarker detection

## Abstract

The Explainable Modular Neural Network (XModNN) enables the identification of biomarkers, facilitating the classification of diseases and clinical parameters in transcriptomic datasets. The modules within XModNN represent specific pathways or genes of a functional hierarchy. The incorporation of biological insights into the architectural design reduced the number of parameters. This is further reinforced by the weighted multi-loss progressive training, which enables successful classification with a reduced number of replicates. The combination of this workflow with layer-wise relevance propagation ensures a robust post hoc explanation of the individual module contribution. Two use cases were employed to predict sex and neuroblastoma cell states, demonstrating that XModNN, in contrast to standard statistical approaches, results in a reduced number of candidate biomarkers. Moreover, the architecture enables the training on a limited number of examples, attaining the same performance and robustness as support vector machine and random forests. The integrated pathway relevance analysis improves a standard gene set overrepresentation analysis, which relies solely on gene assignment. Two crucial genes and three pathways were identified for sex classification, while 26 genes and six pathways are highly important to discriminate adrenergic–mesenchymal cell states in neuroblastoma cancer.

## 1. Introduction

As a subsequent step, the full potential of next-generation sequencing (NGS) assays is being realized for diagnostic purposes through the integration of modern technologies within the context of artificial intelligence (AI). These technologies are being employed as decision support systems. As NGS assays employ high-throughput sequencing technology to sequence the entire genome or transcriptome, issues emerge regarding the identification of specific biomarkers for diseases in the vast array of transcripts and genes. To facilitate clinical cohort studies based on NGS, AI models can be employed to discern between disease types or between healthy and diseased states. Clinical AI models must address issues such as the high complexity of molecular network interactions and the limited number of samples in relation to the numerous input features [1]. Consequently, AI-supported diagnostics on minimal invasive procedures like liquid biopsies have the potential to identify diseases and risk factors without performing invasive biopsies. Such approaches reduce the necessity for invasive interventions and their intrinsic risks, as well as the required resources due to operations. Two main problems in transferring deep learning AI models like neural networks (NNs) into the medical research area are their “black box” nature and the substantial number of examples required for training large models that are needed for complex problems.

To address the issue of “black boxes”, so-called explainable AI (XAI) models can be employed to extract pivotal features from datasets, thereby elucidating the underlying decision-making processes. In general, the application of feature importance weighting allows for the identification of key drivers and biomarkers associated with disease processes. In related research areas, XAI may be employed to uncover phenotypic differences in the skin related to microbiome composition [2] or longitudinal changes in the gut microbiome [3]. An understanding of the decision-making process within an AI model allows for the following benefits: (i) the correction of biases in the training dataset, (ii) an increase in robustness through the highlighting of perturbations, and (iii) the guarantee of meaningful variables for the output [4]. Given the considerable power of AI, it is crucial to ensure that these models are explainable [5] since their complex internal processes are beyond the cognitive reach of humans [6]. Several methods, including Shapley Additive Explanations (SHAP) [7] and Layer-wise Relevance Propagation (LRP) [8], have already been successfully employed to extract informative markers in a robust manner [9] and identify biases in prediction inputs [10] in imaging data. Although the use of LRP for convolutional NNs (CNNs) for post hoc explanation through feature relevance on image datasets is well established [11], its integration with expression datasets remains a relatively novel area of research [12]. The LRP is an explainable AI (XAI) method that backpropagates a single prediction of a neural network through its neurons based on their relevance. This algorithm is a local, pixel-wise decomposition method that adheres to a conservation property, ensuring the redistribution of each neuron’s relevance to lower layers. This approach is suitable for individual diagnoses, analyzing batch effects, and grouping predictive causes in datasets. These datasets typically represent complex biological processes and therefore require many samples for a robust training of standard AI. Additionally, the combination of high interindividual variability in patient expression profiles, batch effects between different cohort studies, and the limitation of examples introduces potential biases in the evaluation process. This can instead result in the classification of artificial confounder features [13] that need to be unveiled.

High-dimensional medical datasets, such as NGS or microarrays, are vulnerable to the “curse of dimensionality”, which can impede performance, leading to unstable interpretability, particularly in the extraction of crucial features. On the one hand, statistical approaches such as Limma [14] or DESeq2 [15] are frequently employed to identify differentially expressed genes (DEGs) in the context of limited replicates [16]. In general, these approaches are effective for identifying potential candidates, but they often result in many candidates, including confounders. Testing a large number of candidates in detail through experiments or conducting extensive literature research to gather information on their molecular functions and pathway integration can be challenging and time-consuming. The combination of statistical approaches and complex AI models like NNs for NGS datasets can be commonly achieved through a preliminary feature selection, which may include tools such as Limma for DEG analysis. Subsequently, the NN can be trained on these smaller pre-filtered datasets of already statistical significant features. This introduces a bias into the analysis, as only a subset of features is utilized due to model limitations. Additionally, less complex interpretable AI models, such as random forests (RF), linear support vector machines (SVMs), and elastic net (ELNET), have been implemented for NGS analysis to extract important features [17]. However, these models are limited in their ability to model biological pathway network interactions, as they focus solely on the gene level and are comparable to statistical DEG tools. Furthermore, a Gene Set Enrichment Analysis (GSEA) [18] is required to facilitate the interpretation of biomarkers within the context of biological processes and pathways. This enables the acquisition of information regarding alterations in homeostasis at a more comprehensive level, as well as insights into the impact of single genes. To address the limitation of AI models requiring a vast number of examples, biologically informed [19] NNs have been developed. These networks utilize a priori information within the model architecture to reduce the number of parameters by assigning neurons in the hidden layer to biological elements, such as genes, and reducing the number of connections between neurons. In the context of molecular reactions and signaling within cells or tissues, modular NNs equipped with a priori knowledge of signaling pathways can be employed to mitigate complexity for an untrained deep NN [20]. Architectures that employ single neurons as pathways of disparate sizes may result in insufficient over-parameterization [21,22]. This forces the model to incorporate biologically irrelevant neurons in order to meet the optimization task introduced as the learning process of the classification task. Moreover, the widely adopted single-loss optimization approach constrains machine learning models’ ability to offer robust explanations for their internal neurons, which in our case are associated with individual pathways. This limitation arises because the optimization process focuses solely on mapping input to output, neglecting the development of stable internal representations. This problem prevents models from selecting robust biomarkers for disease prediction on a local and global interpretation level, which is crucial in the clinical environment or the model explanation.

XModNN addresses these limitations by incorporating the fundamental elements and their interconnections from a functional hierarchy as discrete modules into the architectural framework of an NN. Each module is fully scalable to the complexity of the represented pathway and adapted to the larger structural architecture of XModNN. The weighted multi-loss progressive learning method entails the sequential incorporation of modules comprising weighted losses at varying levels of the hierarchical structure into the training process. This combination results in an intrinsic feature selection, which provides biologically interpretable explanations of the influence of single genes as well as important pathways. The LRP component provides information about local explanations within the network for individual predictions and artifact detection. The aggregation of these explanations across a cohort dataset enables the discovery of biomarkers at the global level. We conducted a benchmarking exercise to evaluate the performance of XModNN in comparison to other AI models, including SVM, RF, and fully connected NN, as well as a statistical approach via Limma. The benchmarking was conducted on an illustrative use case, namely the classification of the proband sex (male or female) in the Study of Health in Pomerania (SHIP) [23]. XModNN inherently linked the crucial gene clusters to their functional pathways, thereby facilitating the robust prediction of the pathway “Ribosome” as a key factor in sex classification. Moreover, XModNN was employed for training on the differentiation of mesenchymal (MES) and adrenergic (ADRN) neuroblastoma cell states using RNA-Seq datasets from the UCSC treehouse database. For the cancer cell state prediction, the pathways “Organismal System (“Taste transduction”, “Sensory System”, “Endocrine System”), “Hippo signaling pathway”, and “Nicotine addiction”, which collectively encompass 26 relevant gene, were identified. In both use cases, XModNN reduced the number of important genes by a factor between 2 and 35 to the other models in both use cases. Furthermore, this eliminated the necessity for an additional GSEA step compared to other approaches and enabled the identification of pathways independent of the number of DEGs by incorporating differences in the expression profiles of all genes within a pathway as an additional parameter.

## 2. Materials and Methods

### 2.1. Assignment of XModNN Modules to KEGG-Brite Hierarchy

To incorporate established external biological insights for the NN architecture, we utilized the KEGG-Brite hierarchy and incorporated it as modules. This functional hierarchy provides comprehensive information on functions related to diseases and drugs, compounds, reactions, and cellular pathways. It consists of four consecutive levels: A, B, C, and D. Level D encompasses in total 17,057 gene entries in the KEGG database for Homo sapiens (https://www.genome.jp/kegg-bin/get_htext?hsa00001.keg (accessed on 24 October 2023). Each of them is subsequently sub-grouped and grouped based on similar pathways and overarching terms creating Level C to A. The input layer of XModNN corresponds to the genes in Level D. In case we have non-unique gene identifier from bead chips or other databases, we used the mean expression across all matching values for the input gene of Level D. Uninformative A-level parts of the KEGG-Brite hierarchy, such as “Brite Hierarchies” and “Not Included in Pathway or Brite”, along with their respective components were excluded and not added as modules in XModNN. Ultimately, this resulted in six A-level groups: ‘Metabolism’, ‘Genetic Information Processing’, ‘Environmental Information Processing’, ‘Cellular Processes’, ‘Organismal Systems’, and ‘Human Diseases’.

For use case 1 (discrimination of sex in whole blood samples), we converted the Illumina probe IDs from the bead chip array of the SHIP-cohort dataset to their corresponding Entrez Gene IDs using the Illumina Bead Chip Product file (https://emea.support.illumina.com/downloads/humanht-12_v3_product_files.html (accessed on 14 November 2023)). We then utilized the KEGG mapper (https://www.genome.jp/kegg/mapper/convert_id.html (accessed on 16 November 2023)) to match them to their respective KEGG identifier and organized the pathways according to the KEGG-Brite hierarchy. This allowed the assignment to Level D and the linkage to the XModNN input layer. In total, we could assign 12,759 Illumina probe IDs to 8043 unique genes at level D in KEGG-Brite. This resulted in six modules representing pathways from level A, 46 modules for level B, and 362 modules for level C. For our use case 2 handling the discrimination of Neuroblastoma cell states, the 58,518 measured mRNAs in the UCSC treehouse dataset (https://treehousegenomics.soe.ucsc.edu/public-data/ (accessed on 27 June 2024)) were annotated to 15,609 Entrez Gene IDs using the R package org.Hs.eg.db (Version: 3.14.0) [24]. We could assign 8137 unique genes at level D in the KEGG-Brite hierarchy.

### 2.2. Optimization, Implementation, Cross-Validation, and Benchmark of Different AI Models

We implemented and optimized XModNN in Python using PyTorch (v1.13.1+cu117). The hyperparameter optimization was performed manually to gain a deeper understanding of the model’s behavior. We evaluated various activation functions, learning rates, batch sizes, and penalty terms, as well as model-specific parameters such as module size, multi-loss weights, or bias disabling for specific layers resulting in parameters that worked well for both use cases. Our model architecture and specialized training procedure directly influence the accumulated gradients calculated by the torch.autograd.backward function for each weight, ensuring the desired capabilities of the model. For XModNN, we used different rules and compared internally different implementations of the LRP ending with the epsilon rule for the best computation (Table 1).

**Figure 1:**
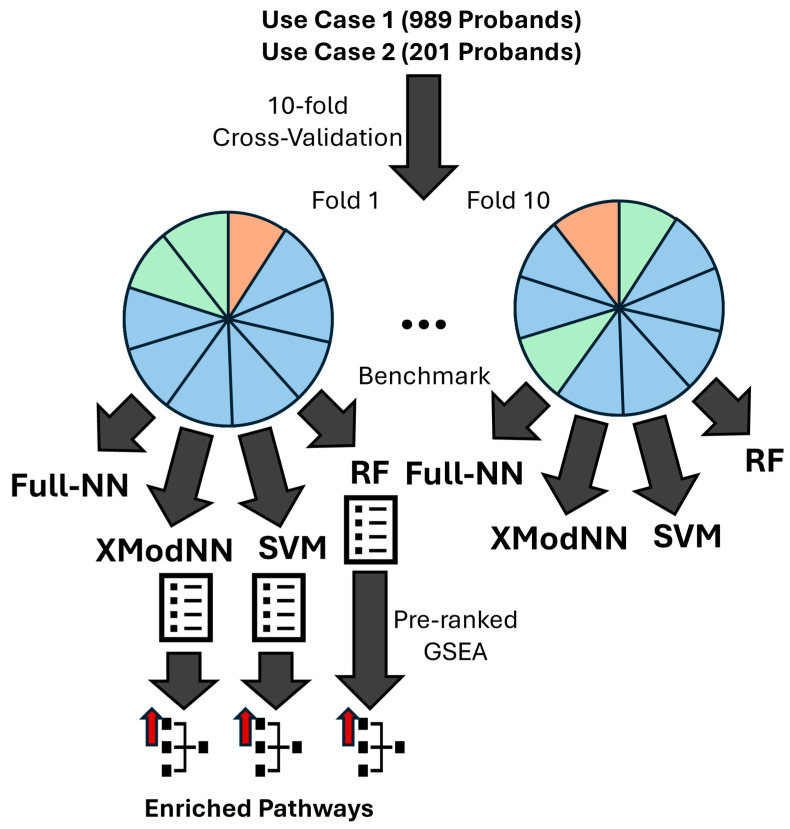
Benchmark overview scheme. The scheme represents, for our use cases, the split for the tenfold cross-validation (test: orange; green: validation; blue: training). The four different models (fully connected NN: Full-NN; XModNN; RF; SVM) are trained, validated (only Full-NN and XModNN), and tested. The important gene lists are used for the pre-ranked GSEA ending up in the prediction of enriched pathways.

For training, validating, and testing our model, we performed a tenfold cross-validation by partitioning the dataset into 10 subsets of nearly equal size, focused on maintaining the original label distribution across each subset (Figure 1). We generated 10 different splits, with each subset serving as the test set once. For the NNs in each split, two randomly selected subsets were designated as the validation set, while the remaining seven subsets were used as the training set.

For benchmarking of XModNN to other AI models, we also implemented a fully connected NN, RF, and a SVM, and used as input the 8043 unique genes at level D in KEGG-Brite for the same training, validation, and test sets. To ensure comparability with XModNN, the fully connected NN with two hidden layers (50 and 10 neurons) used identical regularization methods for benchmarking and was optimized using multiple configurations and hyperparameters. The SVM and RF models were implemented using Scikit-learn (v1.2.1), and the hyperparameter optimization was performed via grid search (GridSearchCV). The SVM was configured with hyperparameters gamma: scale and tol: 0.001, and a linear kernel was chosen for its interpretability. The RF was configured with no specified max_depth and 100 n_estimators. For the random forest (RF) and support vector machine (SVM) models, which do not require validation sets, only the training and test sets were utilized. With this approach, we are able to evaluate the entire dataset, as each element is used once as an unseen prediction. To identify the best model for each cross-validation split, we selected the one with the lowest individual loss. Each selected model was then evaluated using performance metrics, including precision, recall, F1-score, Matthews correlation coefficient (MCC), and balanced accuracy. These models were then utilized for explainability analyses. For standard differential gene expression analysis identifying DEGs, Limma was used as statistical method performed on the gene IDs assigned for use case 1 or use case 2 to the KEGG-Brite hierarchy. Feature relevance for the SVM with a linear kernel was obtained using the model parameters, for the RF by exporting the Scikit-learn feature evaluation, and for the XModNN by applying a custom LRP-method [8,25]. We customized the LRP by normalizing its values during cross-validation to ensure comparability across the tenfold cross-validation process. After aggregating the importance of the last layer for each module for each prediction individually for the entire test set and divided it by the absolute sum of the relevance values of the whole module-layer for normalization. This is possible due to the conservation property of the LRP and does not change the relation between its relevance values. By accumulating the normalized relevance, we can achieve a global explanation for a given dataset. We opted to use the epsilon rule from LRP to incorporate both positive and negative relevance into the normalization process. This sets positive relevance behind a label prediction also in relation to the negative relevance, despite our analysis focusing primarily on positive relevance, to identify those genes important for the decision process.

To identify features of exceptional relevance for the benchmarked models (SVM, RF, and XModNN) and interpret them as potential biomarkers, we calculated gene-wise the mean plus the standard deviation of all relevance values from the test sets from the individual XModNN models and established this as a threshold. Every relevance value of a gene below the mean plus the standard deviation of all relevance values is, in XModNN, interpreted as biomarker of exceptional relevance. Subsequently, we compared these values with the median importance relevance of each feature. This establishes strict, model-specific, and gene-specific thresholds, allowing each model and its associated genes to be individually calibrated. This approach enhances comparability across models and highlights the most relevant features. For the SVM, we utilized the absolute values of the median weights of the models, as they indicate the influence on the classification decision. For the RF, we used the Gini importance as provided by the scikit-learn library. This approach allows us to highlight features across all predicted test sets for further analysis (Figure 1). We used the relevance scores of the genes from every XAI model to perform a pre-ranked GSEA [18]. The GSEA (https://www.gsea-msigdb.org/gsea/index.jsp (accessed on 2 April 2024)) uses the pathways from the KEGG-Brite Hierarchy from the levels A, B, and C and their assignment of genes from level D. Based on this gene to pathway assignment and the pre-ranked relevance gene lists, the GSEA calculates if statistically enriched pathways exist for the different labels of sex (use case 1: male, female) or Neuroblastoma cell state (use case 2: MES, ADRN).

## 3. Results and Discussion

**Figure 2:**
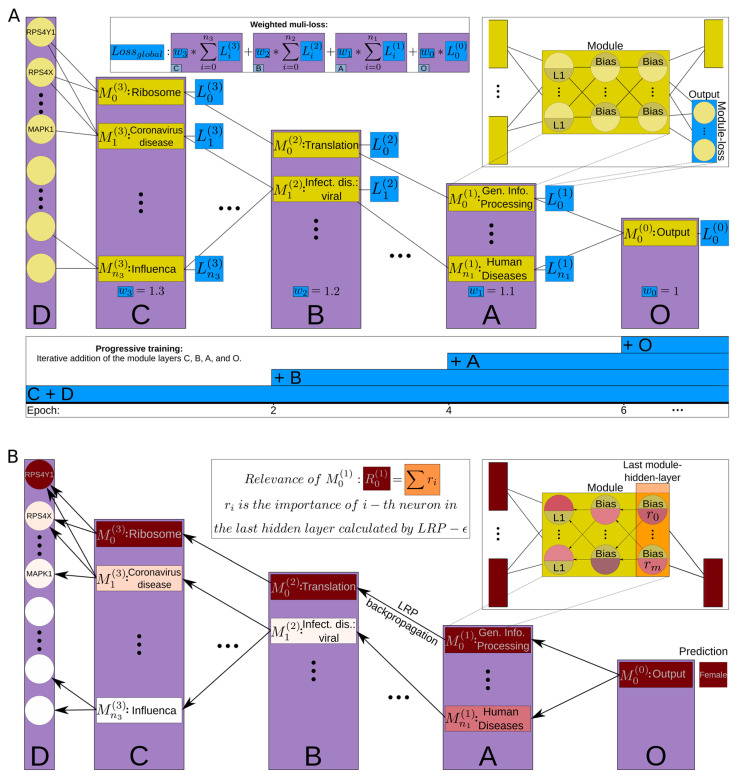
Explainable modular neural network. (**A**) shows the XModNN architecture with the integrated hierarchical structure of the KEGG/Brite hierarchy (levels A to D) to the Output O (purple bars). The yellow modules represent individual pathways/genes. Each module consists of an adaptable NN with its own loss, detailed view at the top right. The weighted multi-loss progressive training is shown in light blue and described in the formula, reflecting sequential addition of weighted losses per layer to the training process. (**B**) illustrates the backpropagation of the custom LRP through XModNN, with relevance represented by a red color gradient.

The distinctive architectural design of XModNN incorporates a biologically informed and modular NN (https://github.com/Wombu/XmodNN (accessible since 19 November 2024)). Each element of a functional hierarchy, such as those in the KEGG, is represented in the architecture by a single module (Figure 2A). Each module is a small neural network (NN) comprising a module-input-layer, one or more tunable module-hidden-layers and a module-output-layer. For both use cases, a fixed-module architecture consisting of three layers, each with three neurons and the tanh activation function, proved sufficient. The module-loss for the module-output-layer is calculated using the weighted cross-entropy function to address class imbalance in the datasets. By employing a more complex module structure, rather than a single neuron as a representative, we reduce the network’s reliance on additional weights and neurons that are unrelated to the biological problem being modeled and are used solely for optimization purposes. The interconnections between the modules are established by linking the final module-hidden-layer with the module-input-layer of the corresponding modules, thereby reflecting connections in the functional hierarchy. The interconnections used to construct XModNN are based on the intersection of the functional hierarchy and the measured values, specifically the measured mRNA intensities from the microarray chip/counts of RNA-Seq and the pathways associated with them. The linkage between the modules is established by multiplying weights and neuron output values, forming the input for neurons in other modules, similar to the connections between neurons in a standard neural network. The module-output-layer is employed for weighted multi-loss progressive training and not connected to other modules. Notable adaptations to the modules include the addition of an L1 penalty term for all individual module-input-layers and the exclusion of the bias term for them, as well as the identity as activation function for the last module-hidden-layer. These promote sparse interactions to enhance selective optimization between modules and improve the quality of the LRP algorithm as an explanatory component [26]. The application of the functional level (A to D) of the KEGG-Brite hierarchy results in a five-layer modular NN. The input layer represents the genes of level D, with gene expression values acting as inputs. The subsequent module-layer represents the modules assigned to the pathways of level C, B, and A of the KEGG-Brite hierarchy (Supplementary Figures S1–S5), while the final module-layer O connects the modules of level A for the classification output.

The training of XModNN is based on a weighted multi-loss progressive training approach. During a mini-batch iteration, the performance of all modules is assessed, resulting in a specific module-loss based on the module-output-layer of the respective module. These are multiplied by loss-weights in accordance with their respective module-layers and aggregated to form the weighted multi-loss (Figure 2A). The assignment of these loss-weights commences at the lowest module-layer C with a value of 1.3 and subsequently decreases by 0.1 in value towards the final module-layer O, reaching 1. These weights can be adjusted during hyperparameter optimization. The objective of this configuration is to mimics the functional hierarchy levels by giving lower module-layer a higher loss-weight. These loss-weight are distinct from those used in the weighted cross-entropy function and are applied subsequent to the calculation of the module-losses. This ensures that XModNN processes information from the features as early as possible while still including influences from other features connected in later module-layers. The conventional approach using a single loss function only establishes a connection between prediction and features without directing the neural network’s internal operations, which are affected by random weight initialization. Using multiple losses ensures a relationship between features and their modules, allowing for a reliable and accurate subsequent analysis. Additionally, this method helps mitigate the “vanishing gradient” problem [27]. The progressive approach starts the training procedure by only optimizing the initial module-layer D and its constituent modules with their respective weighted multi-losses. The subsequent module-layers C to O are then incorporated stepwise into the training process (Figure 2A). This progressive integration of the module-layers can be adjusted in hyperparameter optimization. The later each module-layer is integrated, the more optimized the weights of the previously integrated modules become with a lower module-loss. The selective nature of the modules, combined with the weighted multi-loss and the progressive training steps, create an inherent model-specific feature selection. This approach directly circumvents the curse of dimensionality associated with high-dimensional datasets and allows for a post hoc analysis via LRP for the modules of XModNN (Figure 2B).

### 3.1. Use Case 1: Discriminating the Sex Based on Whole Blood Gene Expression Datasets

The assessment of a biological rationale for clinical parameters or diseases lacks a definitive metric. In order to evaluate the performance of the importance prediction of single genes and pathways, we have elected to utilize a use case example with a relatively straightforward interpretation of important genes. For this reason, we evaluate the explanatory power of XModNN in discriminating between sex (male; female) based on the population-based SHIP [23] dataset, which encompasses a comprehensive range of health-related conditions and is located in the northeastern region of Germany. The SHIP-TREND-0 cohort comprises 4420 participants aged 20 to 79 years, among whom 989 (determined sex: 434 males and 555 females) underwent examination utilizing the Illumina HumanHT-12 v3 bead-chip to obtain gene expression values from whole blood (Supplementary Figure S6). The overall gene expression profiles between male and female subjects exhibited no discernible differences in a straightforward classification task. Additionally, the differentiation across the various pathways and their expression profiles was strikingly similar, not separating the expression profiles for males and females. Within the different pathways of level A in the KEGG-Brite hierarchy, subtle variations in the expression height were observed, with the greatest diversification occurring in the “Genetic Information Processing” pathway (Supplementary Figure S7).

The raw dataset has been processed and normalized according to Homuth et al. (2015) [28]. The log_2_-normalized Omics dataset was mean reduced [29] to minimize the relevance absorption and facilitate a more accurate evaluation with the LRP algorithm [26] and assigned to the KEGG-Brite modules (Supplementary Table S1). For the use case 1, the 989 probands (434 male and 555 female) were for the tenfold cross-validation split in 10 pieces of equal male/female ratio (female: 56%, male: 44%) resulting in ten pieces of nearly equal size (98–100 probands: 5 × 98, 1 × 99, 4 × 100). XModNN demonstrated a pronounced enhancement in the F1-score after ~7 epochs related to the weighted multi-loss progressive training, including, at this stage, the global output module for the prediction (Supplementary Figure S8). In general, the metrics of XModNN demonstrated a robust median balanced accuracy of 1.00. The benchmark comprised the models XModNN, SVM, RF, and a fully connected neural network that were all trained on the same 8043 unique genes at level D in KEGG-Brite.

**Figure 3:**
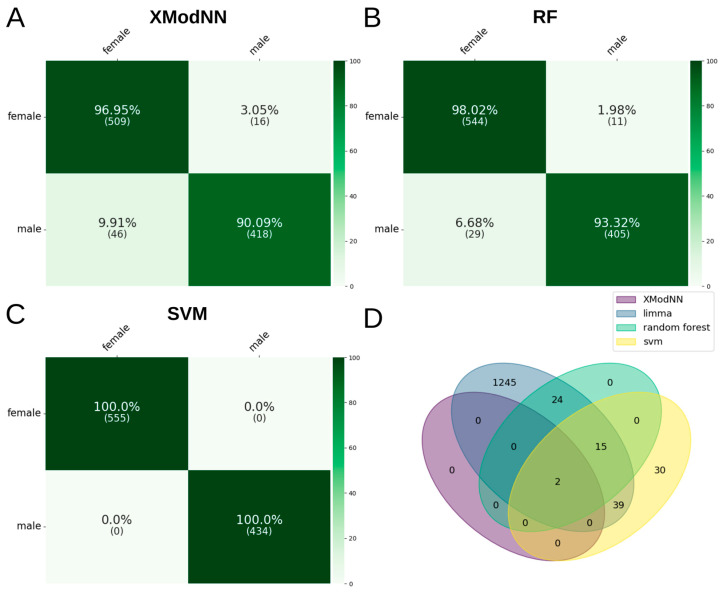
Benchmark for sex prediction. Confusion matrices from the tenfold cross-validation of all combined test sets: (**A**) all XModNN models, (**B**) all SVM models, and (**C**) all RF models. (**D**) A Venn diagram comparing the important genes identified by AI models with those detected by Limma.

In contrast to XModNN, the optimized fully connected neural network was unable to be trained on the SHIP dataset, which may be attributed to the limited number of replicates for the complex dataset. The remaining three methods exhibited a comparable performance in terms of male and female discrimination, as evidenced by the recall and precision values in the confusion matrix (Figure 3A–C).

The SVM demonstrated a perfect accuracy (1.0) in predicting male and female subjects, whereas XModNN and RF exhibited a higher success rate for females (~97.5%) than males (~91.5%). The benchmark of XModNN, RF, and SVM demonstrated comparable mean performance in terms of F1 (~0.94/~0.96/1.00) and balanced accuracy scores (~0.94/~0.96/1.00, Table 2). The lower mean scores of XModNN are attributable to one poorly performing model, as evidenced by the low minimum scores, while achieving perfect median scores of 1.00.

**Figure 4:**
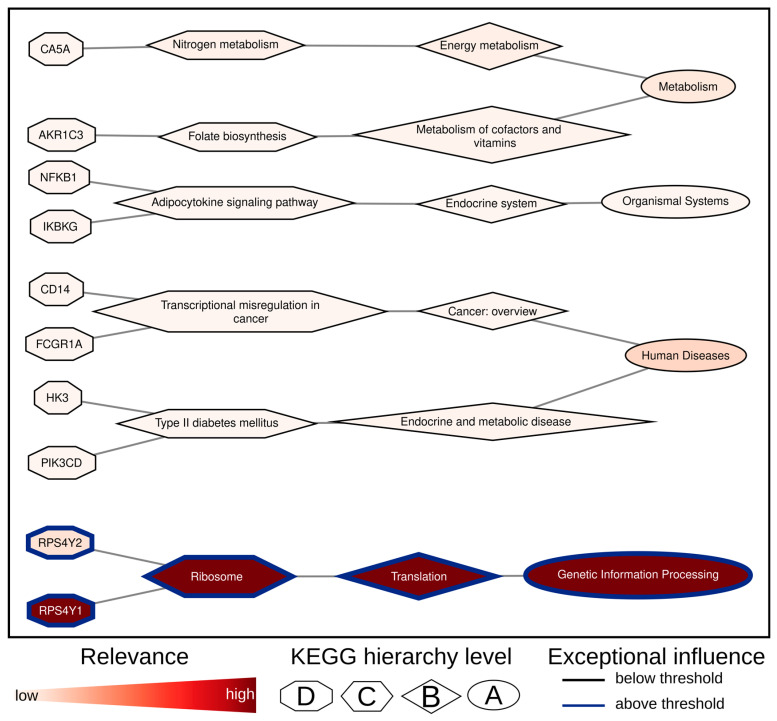
Relevance network for sex discrimination using XModNN. This network created with Cytoscape illustrates the identified relevant genes and pathways based on the XModNN output for the sex use case on the SHIP dataset, with node shapes indicating KEGG hierarchy levels A, B, C, and D. Genes and pathways outlined in blue are deemed exceptionally relevant based on the XModNN threshold. The color gradient within the nodes reflects the calculated XModNN relevance, ranging from white (irrelevant, low) to red (relevant, high).

In order to evaluate the relevance of the genes in question with regard to the specified use case, we conducted a comparison of the overlap between the AI models and the DEGs from Limma (1325 DEG; Supplementary Table S2). In general, 99 of the 1325 DEGs have been identified as relevant for discriminating between male and female using SVM, RF, or XModNN. The genes RPS4Y1 and RPS4Y2 were identified as being present in the overlap between the XModNN, RF, and SVM models (Supplementary Tables S3–S5). The SVM model identified 86 genes as important, whereas the RF reduced this number to 41 and XModNN to two candidates, in accordance with the strict cutoff threshold (Figure 3D). In addition to the identification of crucial genes for discrimination, it is also essential to comprehend the direct and indirect context of regulatory processes and pathways, as this facilitates the elucidation of the underlying mechanisms. In order to identify enriched pathways, an additional pre-ranked GSEA must be performed for the AI models and Limma (Supplementary Tables S6–S8). One disadvantage of this process is that it is dependent on the identified important genes alone. In contrast, XModNN performs an enhanced pre-ranked GSEA directly within the model architecture (Supplementary Tables S9–S11), evaluating the importance of all genes simultaneously at the various hierarchical levels (Figure 4). In total, 26 (20 female, 6 male) enriched pathways were identified with the bead chip array in the context of the use case of sex using the GSEA on the DEGs of Limma. Based on the DEGs of Limma, only the “Ribosome” pathway was enriched with a positive foldchange for classifying sex, including the ribosomal proteins of the small subunit 4 located on the Y-chromosome (RPS4Y1, RPS4Y2). The same results could be observed in the evaluation of XModNN (Figure 4). In contrast, no enriched pathways could be detected using the important genes from the SVM. The RF showed “signal transduction”, ”organismal systems”, “PI3K-AKT signaling pathway”, “cellular community”, and “human papillomavirus infection” as enriched pathways. Interestingly, these pathways do not include RPS4Y1 and RPS4Y2, which are the common important genes within all XAI models.

The relevance analysis conducted using XModNN aligned with the results obtained from Limma, particularly regarding the enriched “Ribosome” pathway, which includes the genes RPS4Y1 and RPS4Y2. In agreement with the Limma findings, XModNN identified the “Ribosome” pathway as significant while deeming the “Corona Disease” pathway unimportant, despite both pathways containing RPS4Y1 and RPS4Y2. Unlike Limma’s GSEA, XModNN recognized the broader category of “Genetic Information Processing” as important, taking into account not only the number of genes within a pathway but also the connections within the hierarchical structure. In conclusion, XModNN demonstrated a performance comparable to simpler explainable AI models, achieving an F1 score of approximately 1.00, while surpassing the performance of a non-trainable fully connected neural network. This indicates that XModNN can be effectively trained on small sample sizes while remaining a scalable, complex model capable of addressing intricate problems. Moreover, XModNN reduced the number of potential biomarkers by a factor of approximately 650 compared to those identified by Limma while still identifying the same significant pathways as those determined by GSEA based on Limma’s results. The advantage of XModNN’s intrinsically enhanced Gene Set Overlap Analysis (GSOA) becomes evident when compared to the GSEA conducted on the important genes identified by SVM and RF. In these cases, the pathways generated were often uninterpretable, as they did not take the hierarchical structure into account, focusing instead on independent pathways.

### 3.2. Use Case 2: Discriminating Neuroblastoma Cancer Cell States

Next, we demonstrated the potential of XModNN to extract the most important biomarker to discriminate between diseases or, in the present case, between neuroblastoma cancer cell states (mesenchymal, MES; adrenergic, ADRN). In order to evaluate the efficacy of predicting the importance of individual genes and pathways, a well-studied dataset from the UCSC Treehouse and the published discrimination between MES and ADRN neuroblastoma were employed [30]. In accordance with the aforementioned publication, the differentiation between MES and ADRN is based on the expression of 460 and 346 genes. Consequently, the explanatory power of XModNN in discriminating between MES and ADRN neuroblastoma is evaluated using the sample labeling from Mabe et al. (2022) [30]. The 201 samples from the UCSC Treehouse dataset were labeled according to the MES and ADRN neuroblastoma cell states, resulting in 173 ADRN and 28 MES samples for the same 8043 unique genes at level D in KEGG-Brite. The ratio of MES to ADRN participants per piece was approximately 14% MES to 86% ADRN, resulting in dataset pieces of sizes between 19 and 21 (1 × 19; 6 × 20; 3 × 21). The dataset comprises poly-A purified total RNA-Seq, transformed by log_2_(x + 1) using transcripts per million mapped reads (TPM) and quantified by RSEM (RNA-Seq by Expectation-Maximization) [31]. Upon examination of the various pathways within the KEGG-Brite hierarchy at the level A, subtle variations in expression height and form were observed (Supplementary Figure S9). In particular, significant differences were observed in the mean expression and density of genes involved in Organismal Systems and Genetic Information Processing.

XModNN showed a pronounced improvement in F1 score after ~6–7 epochs related to the weighted multi-loss progressive training during the tenfold cross-validation, comparable to use case 1 (Supplementary Figure S10). In general, the metrics of XModNN showed a robust median balanced accuracy above 0.97, and the benchmark to RF and SVM showed a comparable overall prediction accuracy (Table 3). Notably, RF and SVM exhibited substantially lower minimum balanced accuracy scores (0.47/0.67, respectively), whereas the least effective model in XModNN still achieved relatively strong performance, with a balanced accuracy of 0.80.

**Figure 5:**
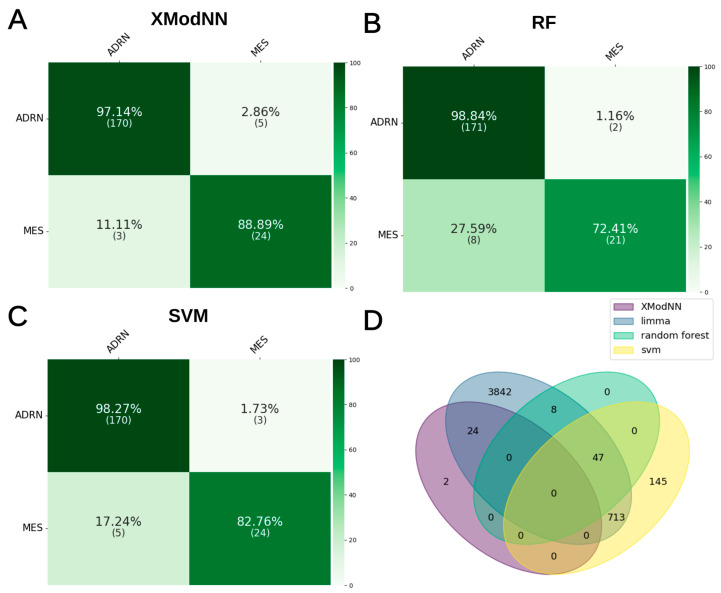
Benchmark of Neuroblastoma cell state prediction. Confusion matrices from the tenfold cross-validation of all combined test sets: (**A**) all XModNN models, (**B**) all SVM models, and (**C**) all RF models. (**D**) A Venn diagram comparing the important genes identified by AI models with those detected by Limma.

**Figure 6:**
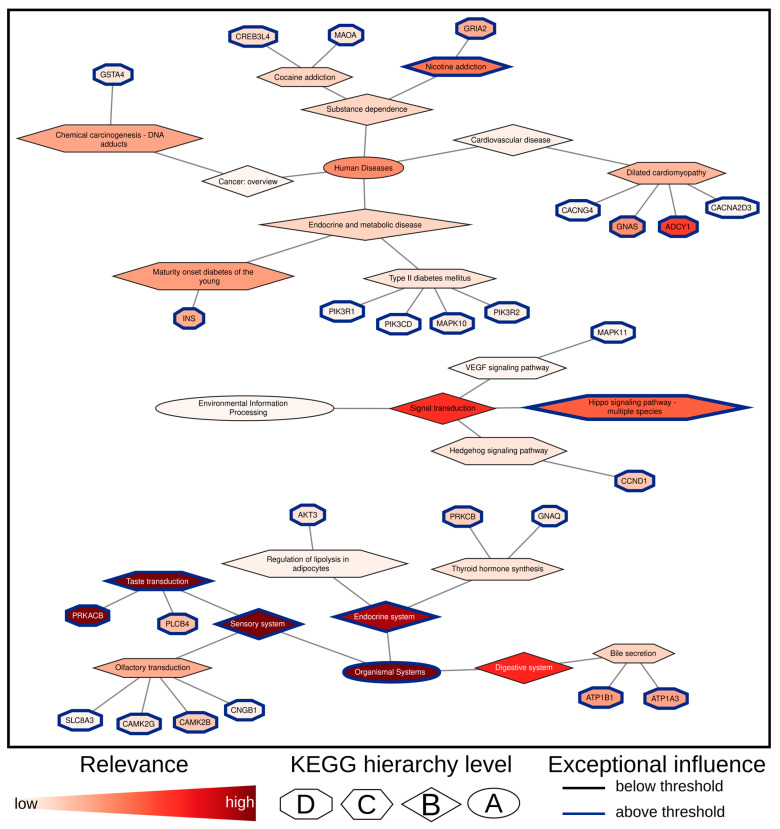
Relevance network for of Neuroblastoma cell state discrimination using XModNN. This network created with Cytoscape illustrates the identified relevant genes and pathways based on the XModNN output for the neuroblastoma use case, with node shapes indicating KEGG hierarchy levels A, B, C, and D. Genes and pathways outlined in blue are deemed exceptionally relevant based on the XModNN threshold. The color gradient within the nodes reflects the calculated XModNN relevance, ranging from white (irrelevant, low) to red (relevant, high).

In contrast to the first use case involving sex discrimination, a higher false-positive rate was observed for the SVM and RF models in this analysis (Figure 5A–C). Furthermore, Limma identified a threefold increase in differentially expressed genes (DEGs), totaling 4634. Notably, none of the key genes identified through the Limma analysis were consistently detected across all three AI models (Figure 5D). Specifically, while 24 of the 26 genes identified by XModNN (including PRKACB, ADCY1, GNAS, ATP1A3, GRIA2, INS, PLCB4, CCND1, CAMK2B, PRKCB, CREB3L4, CAMK2G, AKT3, GNAQ, GSTA4, MAPK10, PIK3R2, CNGB1, PIK3R1, CACNA2D3, MAPK11, CACNG4, and SLC8A3) were found to overlap with Limma, there was no overlap with either SVM or RF models, aside from the specific relevant genes ATP1B1 and PIK3CD. Conversely, SVM and RF exhibited considerable overlap with each other and with Limma (Supplementary Tables S12–S15). The 26 significant genes identified by XModNN correspond to three distinct level A pathways, demonstrating a broader range of normalized importance compared to the first use case involving sex discrimination (Figure 6). This variance may be attributed to increased interindividual variability.

Comparing the relevant gene lists from Mabe et al. (2022) [30] with those identified by XModNN reveals that the most significant genes from XModNN do not overlap with the list of Mabe et al. (Supplementary Table S16). Only ATP1B1 (MES), GRIA2, CCND1, and PIK3R1 (all ADRN) were found in both lists. Nevertheless, XModNN incorporated 101 genes associated with ADRN and 76 genes associated with MES from the Mabe et al. (2022) gene lists for classification, with a median relevance score greater than 0.00. For nearly all of the 26 relevant genes identified by XModNN not listed by the publication, the existing literature establishes a connection to cancer, although not necessarily specific to Neuroblastoma. This observation may suggest that these important genes are not strictly linked to a specific cell state but are instead relevant to Neuroblastoma more broadly, indicating that their combination may be significant for discrimination. This aligns with the definition of labels based on the mean activation of gene sets. Notably, there was a reduction of approximately 180-fold in the number of putative biomarkers needed to identify MES from ADRN compared to those identified by Limma.

XModNN identified additional pathways (Supplementary Tables S17–S19) that are significant for distinguishing between ADRN and MES cell states, indicating potential new targets for therapeutic strategies at the specific gene level (Supplementary Tables S20–S21). Pathways such as “Organismal Systems” (including “Taste Transduction”, “Sensory System”, and “Endocrine System”), the “Hippo Signaling Pathway”, and “Nicotine Addiction” have already been linked to cancer and Neuroblastoma [32]. Notably, unique genes associated with MES cells indicate their stem-like properties and resistance to therapy. The MES biomarker identified by XModNN is primarily associated with resistance, and several of these targets are potentially druggable. The discovery of genes that highlight the stem-like and therapy-resistant characteristics of MES cells underscores the clinical challenges in treating these tumors. A better understanding of the heterogeneity in gene expression profiles across cancer cell states could provide deeper insights into the intra-tumoral heterogeneity of Neuroblastoma, complementing existing approaches focused on super-enhancers and transcription factor networks. The novel markers identified by XModNN may aid in refining diagnostic and treatment strategies, particularly in elucidating the mechanisms of chemoresistance associated with MES cells. The important genes identified may represent downstream effects or alternative regulatory pathways not captured by super-enhancer analyses, offering a broader perspective on the differentiation states of Neuroblastoma and their clinical implications. Further validation and functional studies of these genes are essential to clarify their roles and therapeutic potential in Neuroblastoma.

The benchmark against statistical linear and non-linear models like Limma showed the potential of explainable AI models to predict and reduce the number of potential biomarkers, especially XModNN. Statistical models evaluate each gene individually by creating separate linear models, without accounting for potential gene–gene interactions. In contrast, XModNN, as well as RF and SVM, evaluate genes collectively, allowing for a substantial reduction in the number of relevant features. Specifically, XModNN can reduce the number of relevant genes even further due to its higher model complexity and biologically informed structure, enabling it to adapt to complex dataset structures more effectively. This work demonstrates that XModNN is a specialized algorithm optimized for performance with limited datasets. Built upon NN architecture, XModNN benefits from a robust theoretical foundation and wide adaptability, allowing for it to be readily applied across diverse datasets, including those with multiple labels or variable input values. The combination of progressive multi-loss training and a modular design enables the model to adjust its components to align with the structure of the specific dataset. Furthermore, XModNN provides both local and global interpretability, supporting applications in personalized medicine, confounder and bias detection, and biomarker discovery.

## Figures and Tables

**Table 1:** Optimized hyperparameters for XModNN. This Table contains the optimized hyperparameter for the fully connected NN and XModNN for the use cases 1 (Sex) and 2 (Neuroblastoma cell state).

Parameter	Sex	Neuroblastoma Cell State
Module size	3, 3, 3 + module-output layer	3, 3, 3 + module-output layer
Weight initialization	Normalized Xavier	Normalized Xavier
Activation Function	tanh	tanh
Learning rate	0.01	0.01
Mini batch size	32	16
Loss	Weighted cross-entropy loss	Weighted cross-entropy loss
Class weights for loss	automatic	automatic
Penalty	L1	L1
Penalty multiplikator all weights	0.01	0.01
Penalty multiplikator first module-hidden layer	0.1	0.1
Disable Bias: first module-hidden layer	True	True
Disable Bias: module-output layer	True	True
Weighted multi-loss progressive Training		
Muli-loss weights	C: 1.3, B: 1.2, A:1.1, O:1	C: 1.3, B: 1.2, A:1.1, O:1
Progressive module-layer inclusion	B: 2, A: 4, O: 6	B: 2, A: 4, O: 6

**Table 2:** Performance metrics of benchmark of XAI models for use case 1. The performance metrics are organized for XModNN/RF/SVM and listed for median, mean, standard deviation (Std.), minimum (Min.), and maximum (Max.) based on the best model each of the tenfold cross-validation on the test set.

	Median	Mean	Std.	Min.	Max.
F1-Score	1/0.96/1	0.94/0.96/1	0.1/0.01/0	0.74/0.94/1	1/0.99/1
Balanced accuracy	1/0.95/1	0.94/0.96/1	0.1/0.01/0	0.74/0.94/1	1/0.99/1
MCC	1/ 0.91/1	0.87/0.92/1	0.2/0.03/0	0.47/0.88/1	1/0.98/1

**Table 3:** Performance metrics of benchmark of XAI models for Neuroblastoma cell states MES and ADRN (use case 2). The performance metrics are organized for XModNN/RF/SVM and listed for median, mean, standard deviation (Std.), minimum (Min.), and maximum (Max.) based on the best model each of the tenfold cross-validation on the test set.

	Median	Mean	Std	Min	Max
F1-score	0.91/0.94/0.96	0.92/0.87/0.91	0.07/0.17/0.10	0.80/0.44/0.72	1/1/1
Balanced accuracy	0.97/0.92/0.99	0.97/0.86/0.91	0.08/0.17/0.11	0.80/0.47/0.67	1/1/1
MCC	0.84/0.90/0.92	0.85/0.76/0.84	0.14/0.33/0.17	0.61/0.00/0.55	1/1/1

## Data Availability

The source code as well as readme and test datasets are available as github repository at: https://github.com/Wombu/XModNN (accessible since 19 November 2024).

## Supplementary Materials

The following supporting information can be downloaded at: https://www.mdpi.com/article/10.3390/biom14121501/s1, Figure S1: KEGG hierarchy visualization Level D1; Figure S2: KEGG hierarchy visualization Level D2; Figure S3: KEGG hierarchy visualization Level C; Figure S4: KEGG hierarchy visualization Level B; Figure S5: KEGG hierarchy visualization Level A; Figure S6: Gene Expression Distribution Across Sexes; Figure S7: Comparison of gene expression across Sexes by KEGG-Brite level A; Figure S8: Performance metrics from 10-fold cross-validation fo sex; Figure S9: Comparison of gene expression across Neuroblastoma sub-types by KEGG-Brite level A; Figure S10: Performance metrics from 10-fold cross-validation for Neuroblastoma sub-types. Table S1: KEGG-Brite Hierarchy modules in XModNN; Table S2: DEGs from Limma for Use case 1; Table S3: Relevant Genes from XModNN for Use case 1; Table S4: Important Genes from RF for Use case 1; Table S5: Important Genes from SVM for Use case 1; Table S6: Enriched Pathways from GSEA for Limma; Table S7: Enriched Pathways from GSEA for RF; Table S8: Enriched Pathways from GSEA for SVM; Table S9: Relevant Pathways level C for XModNN in Use Case 1; Table S10: Relevant Pathways level B for XModNN in Use Case 1; Table S11: Relevant Pathways level A for XModNN in Use Case 1; Table S12: DEGs from Limma for Use case 2; Table S13: Relevant Genes from XModNN for Use case 2; Table S14: Important Genes from RF for Use case 2; Table S15: Important Genes from SVM for Use case 2; Table S16: Signature Genes for MES and ADRN based on Mabe et al. 2022; Table S17: Relevant Pathways level C for XModNN in Use Case 2; Table S18: Relevant Pathways level B for XModNN in Use Case 2; Table S19: Relevant Pathways level A for XModNN in Use Case 2; Table S20: XModNN relevance level D (genes) for ADRN label training; Table S21: XModNN relevance level D (genes) for MES label training.
