# Supplementary material for: XModNN: Explainable Modular Neural Network to Identify Clinical Parameters and Disease Biomarkers in Transcriptomic Datasets
Source: Biomolecules. 2024 Nov 25;14(12):1501. doi: 10.3390/biom14121501 (PMC11673716; doi:10.3390/biom14121501)

Supplemental Figure S1-S5 KEGG hierarchy visualization. This figure illustrates the hierarchical structure of KEGG-Brite pathways. The y-axis represents level C pathways, and the x-axis represents level B pathways. Each marker indicates the inclusion of level C pathways within their respective level B pathways. The color gradient corresponds to level A pathways, while the numbers within the markers denote the count of level D gene expression values associated with each level C pathway.

Supplemental Figure S6: Gene Expression Distribution Across Sexes. This violin plot illustrates the distribution of gene expression levels between sexes for the gene expression dataset from the SHIP study.

Supplemental Figure S7: Comparison of gene expression across Sexes by KEGG-Brite level A. These violin plots compare gene expression levels between sexes, stratified according to Level A of the KEGG-Brite hierarchy.

Supplemental Figure S8: Performance metrics from 10-fold cross-validation fo sex. This figure presents the performance metrics evaluated during the 10-fold cross-validation training process. Section A visualizes cross-entropy loss across all models, emphasizing the effect of weighted multi-loss progressive training, where layers are incrementally added. Section B shows the F1-score, which balances precision and recall, to demonstrate model performance improvement during training. Additionally, the training progression of the best-performing model from the cross-validation is depicted, offering detailed insight into its learning behaviour.

Supplemental Figure S9: Comparison of gene expression across Neuroblastoma sub-types by KEGG-Brite level A. These violin plots compare gene expression levels between Neuroblastoma sub-types, stratified according to Level A of the KEGG-Brite hierarchy.

Supplemental Figure S10: Performance metrics from 10-fold cross-validation for Neuroblastoma sub-types. This figure presents the performance metrics evaluated during the 10-fold cross-validation training process. Section A visualizes cross-entropy loss across all models, emphasizing the effect of weighted multi-loss progressive training, where layers are incrementally added. Section B shows the F1-score, which balances precision and recall, to demonstrate model performance improvement during training. Additionally, the training progression of the best-performing model from the cross-validation is depicted, offering detailed insight into its learning behaviour.

Sup. Fig. S1: KEGG hierarchy visualization

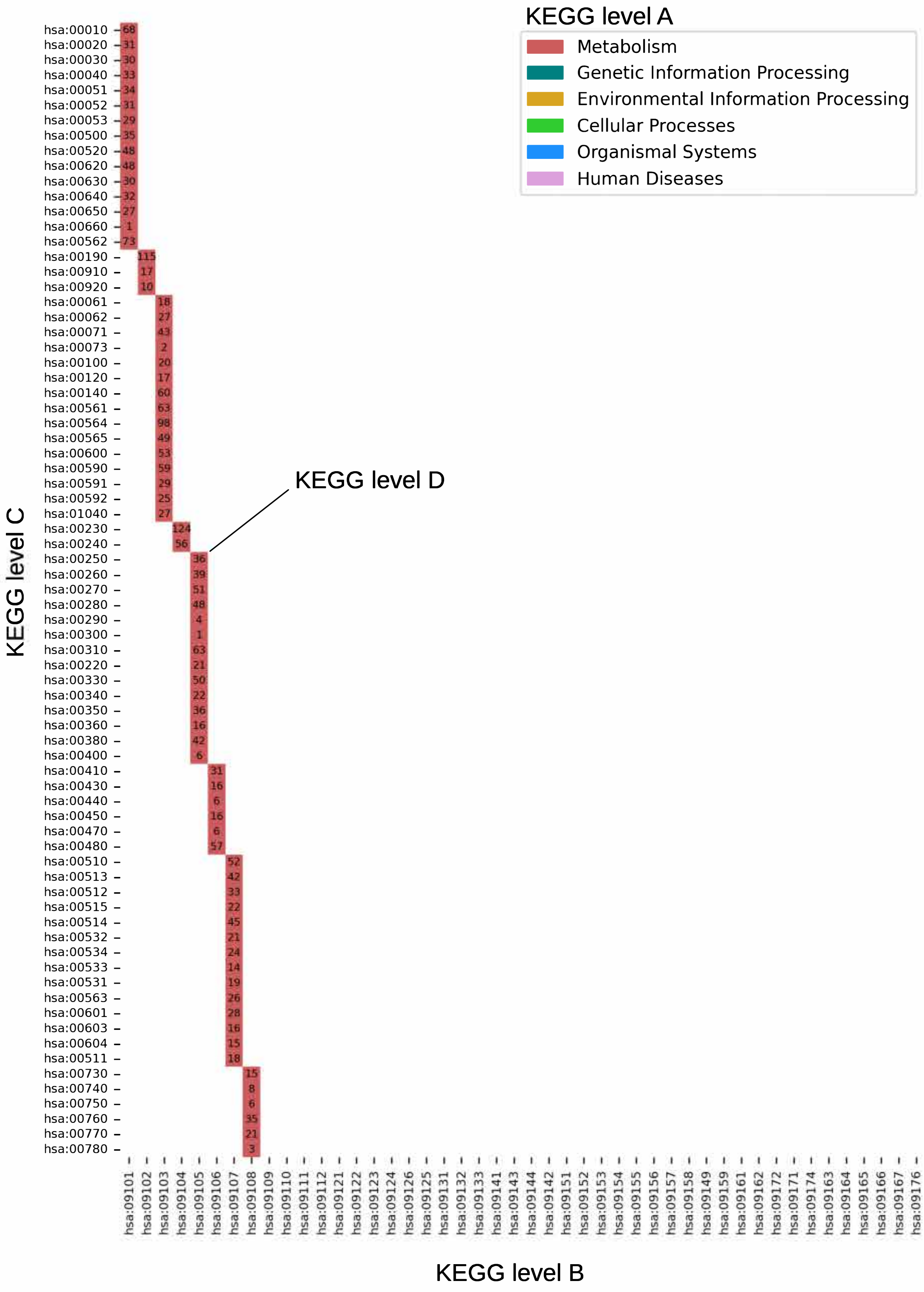

Sup. Fig. S2: KEGG hierarchy visualization

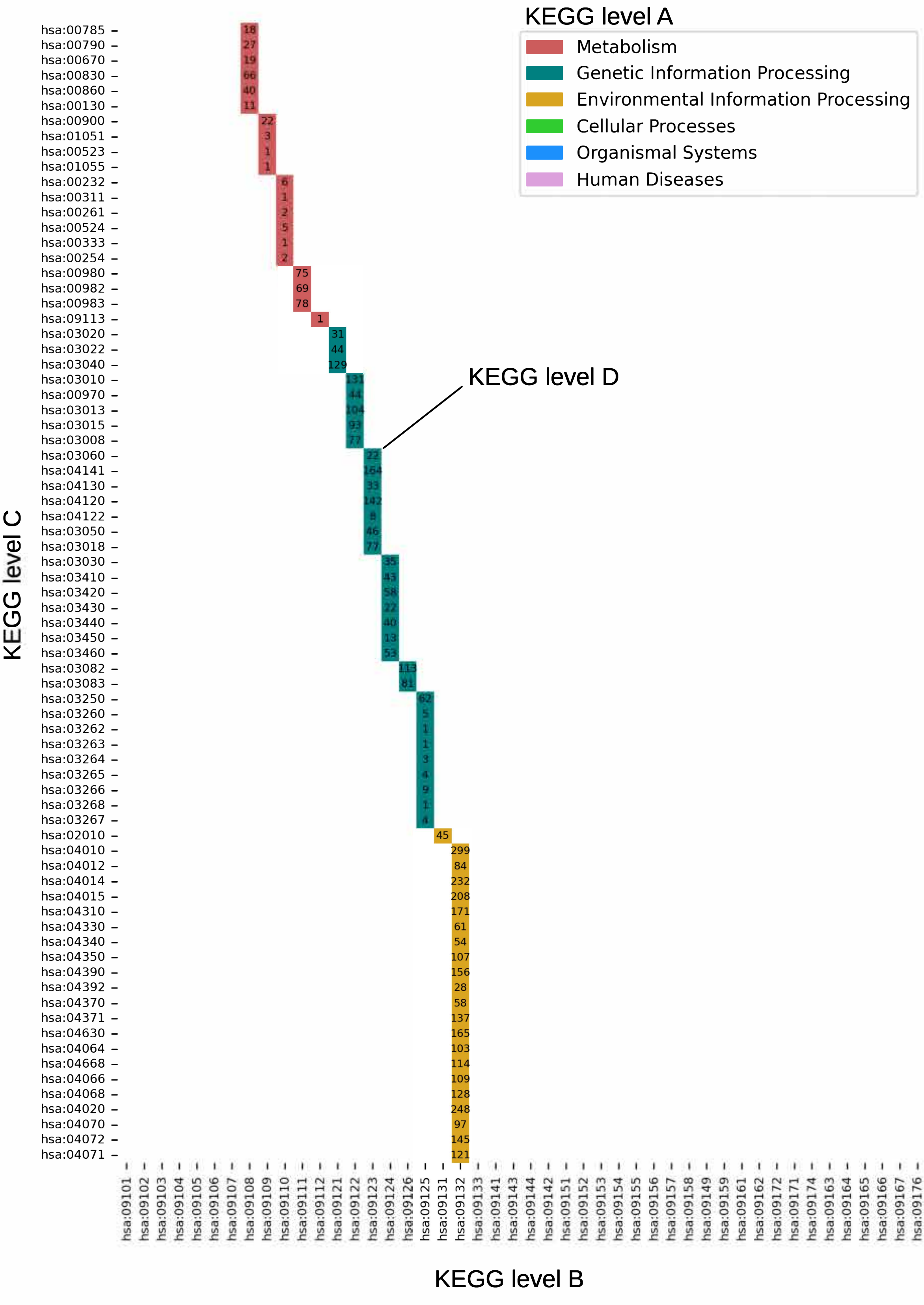

Sup. Fig. S3: KEGG hierarchy visualization

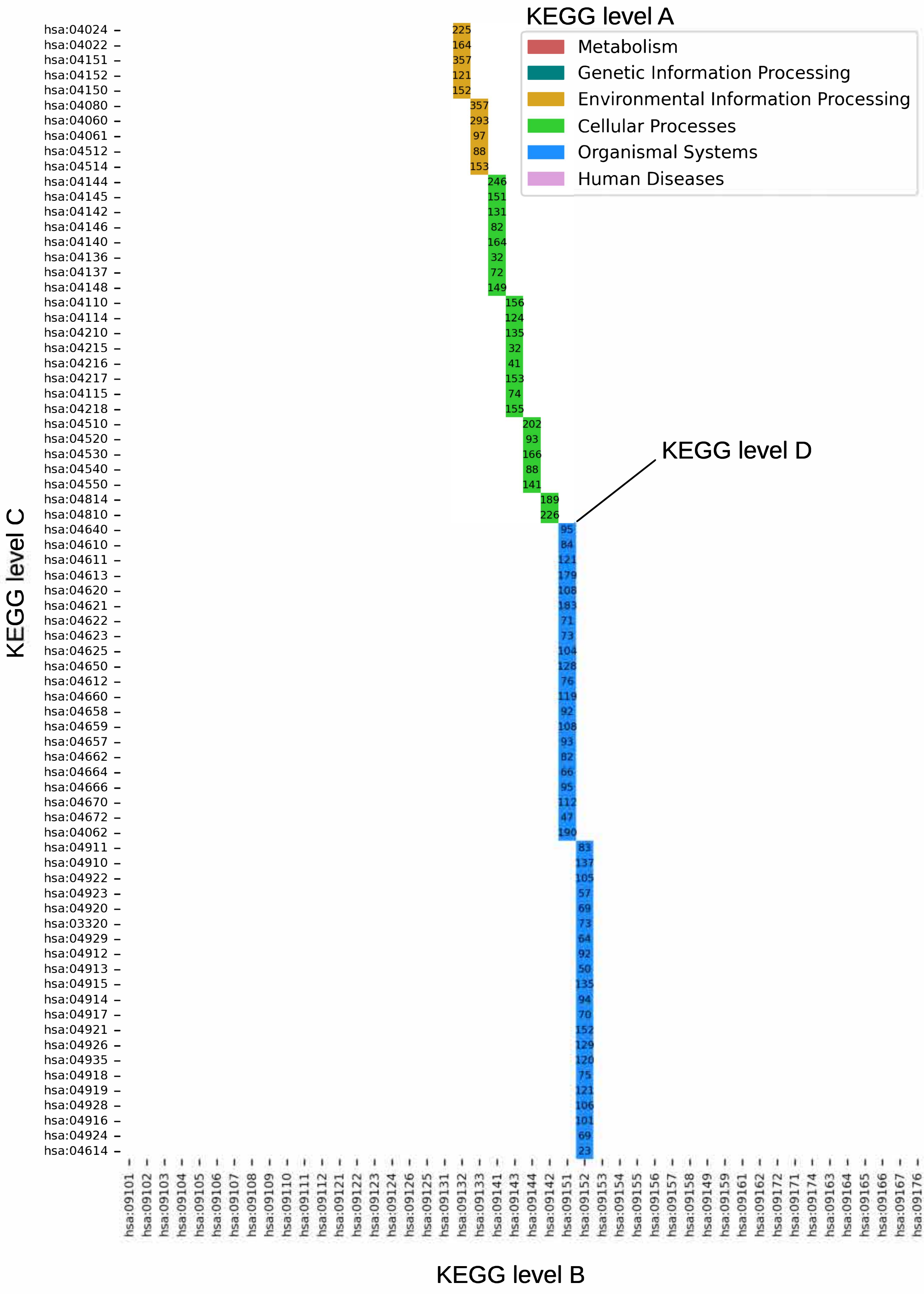

Sup. Fig. S4: KEGG hierarchy visualization

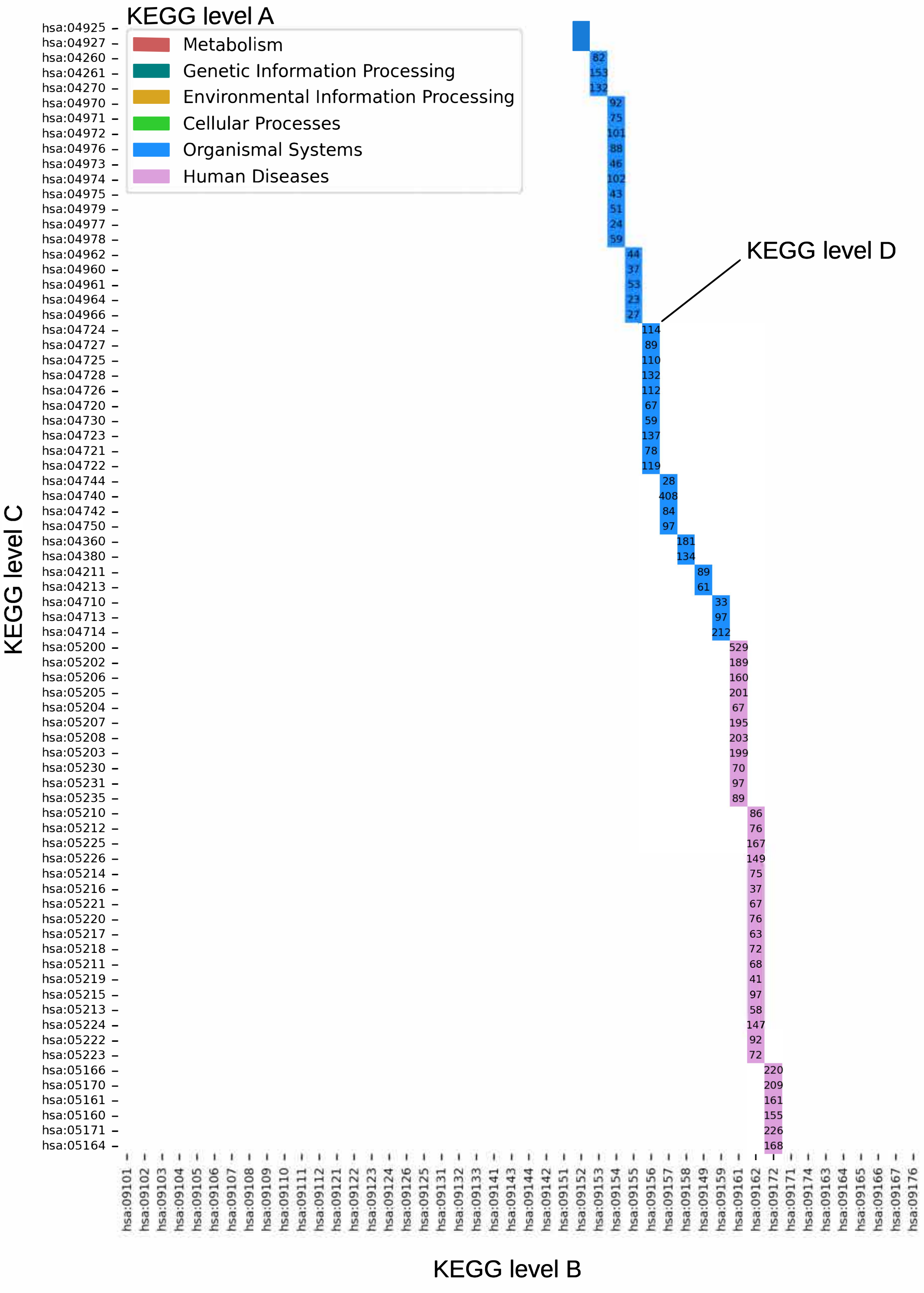

Sup. Fig. S5: KEGG hierarchy visualization

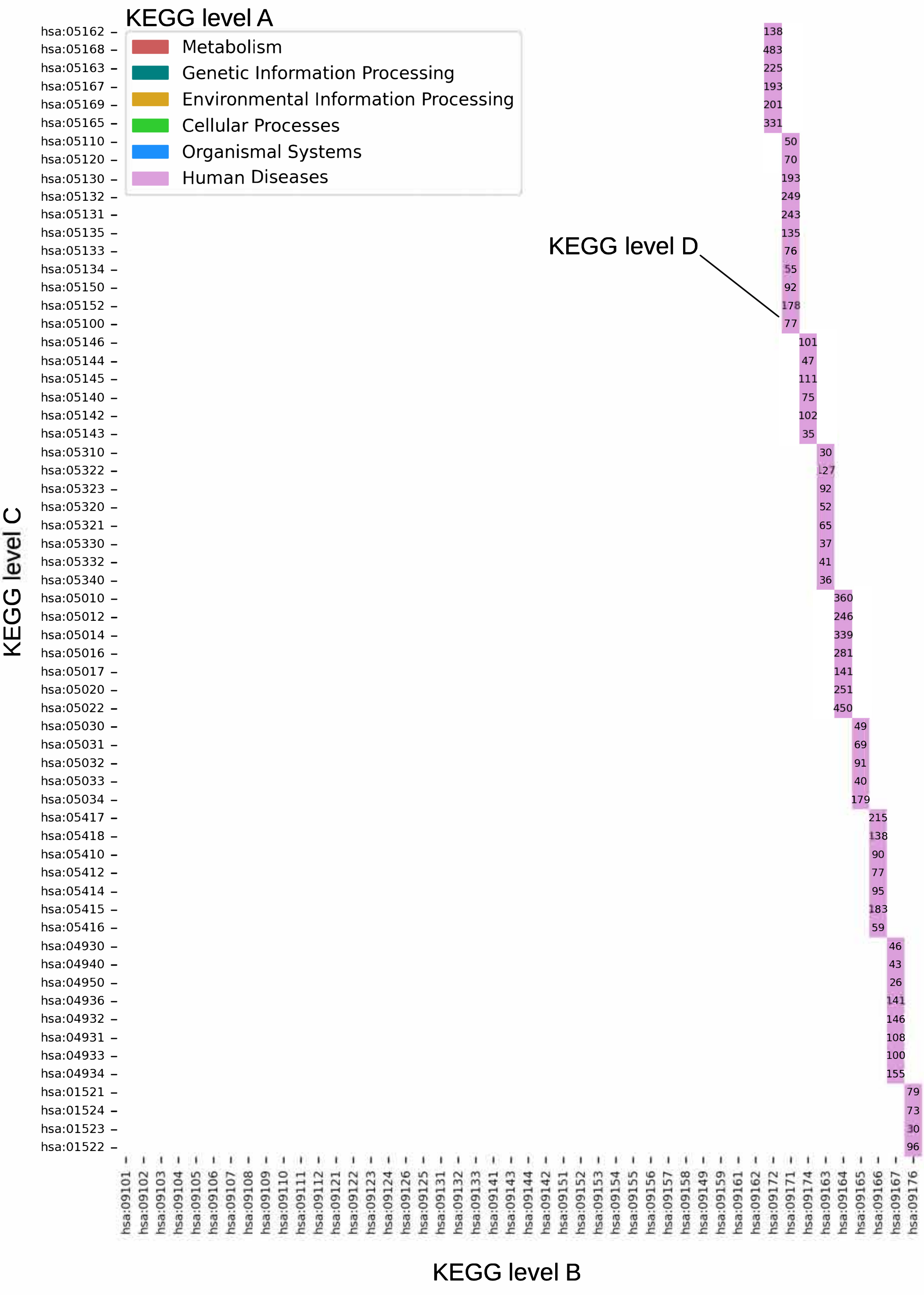

Sup. Fig. S6: Gene Expression Distribution Across Sexes

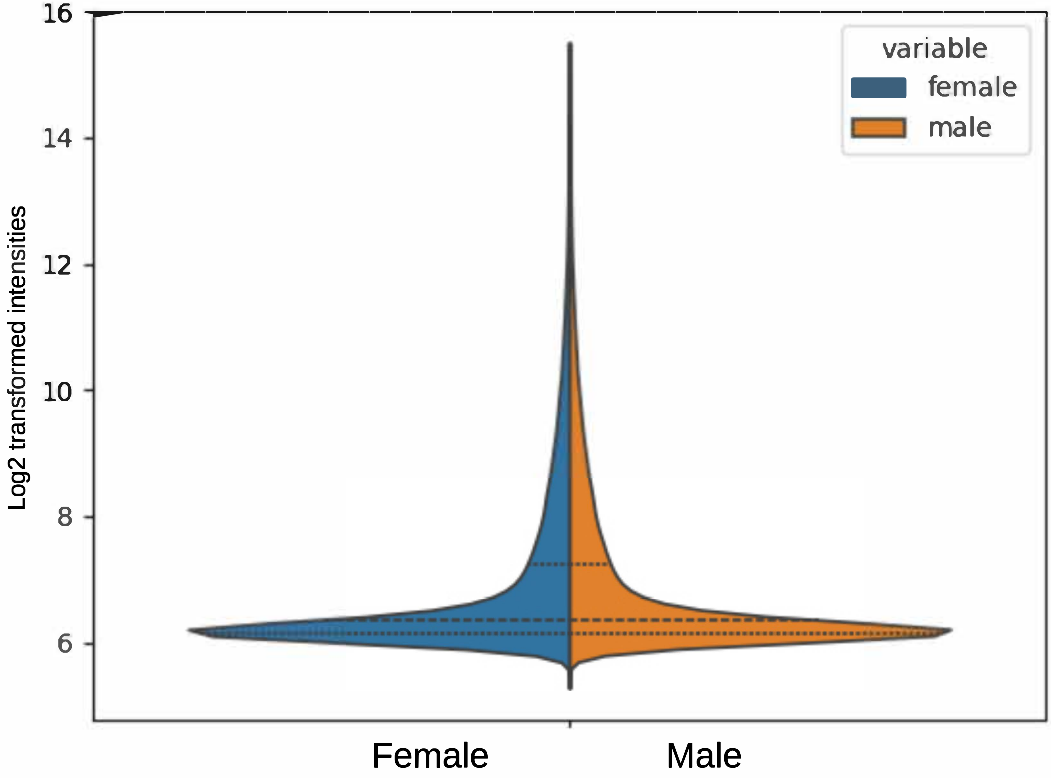

Sup. Fig. S7:Comparison of gene expression across Sexes by KEGG-Brite level A

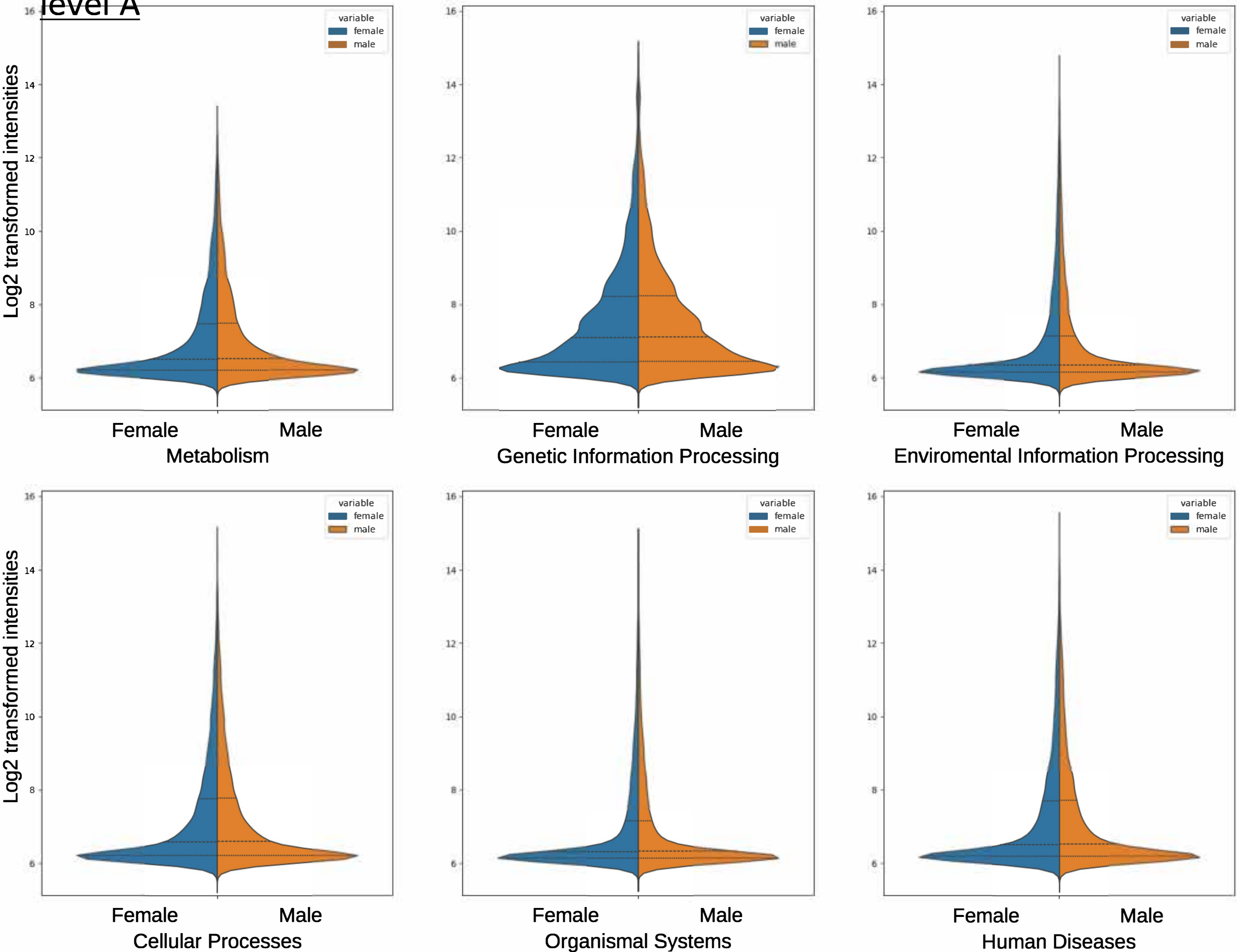

Sup. Fig. S8. Performance metrics from 10-fold cross-validation for sex

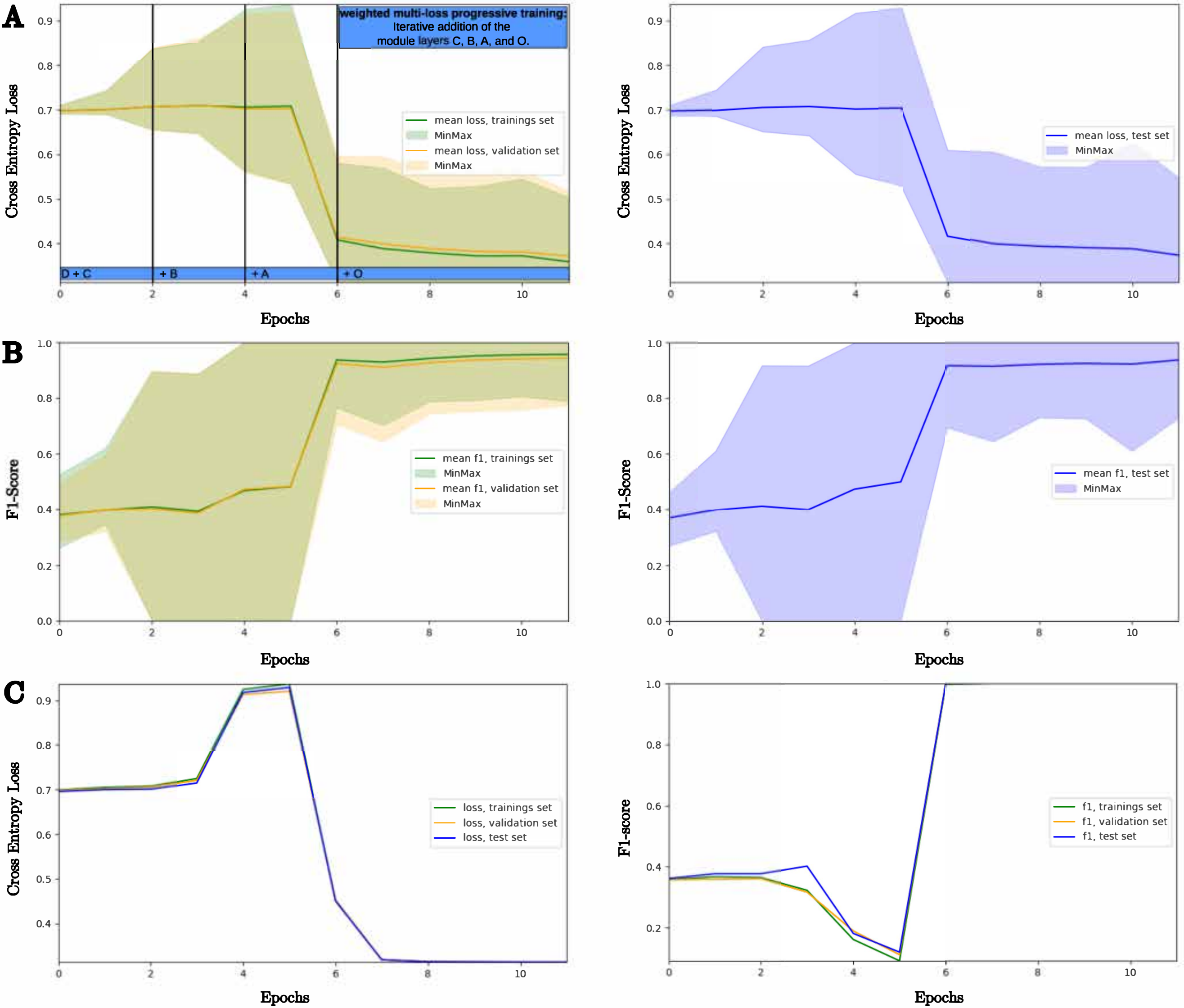

Sup. Fig. S9. Comparison of gene expression across Neuroblastoma sub-types by KEGG-Brite level A

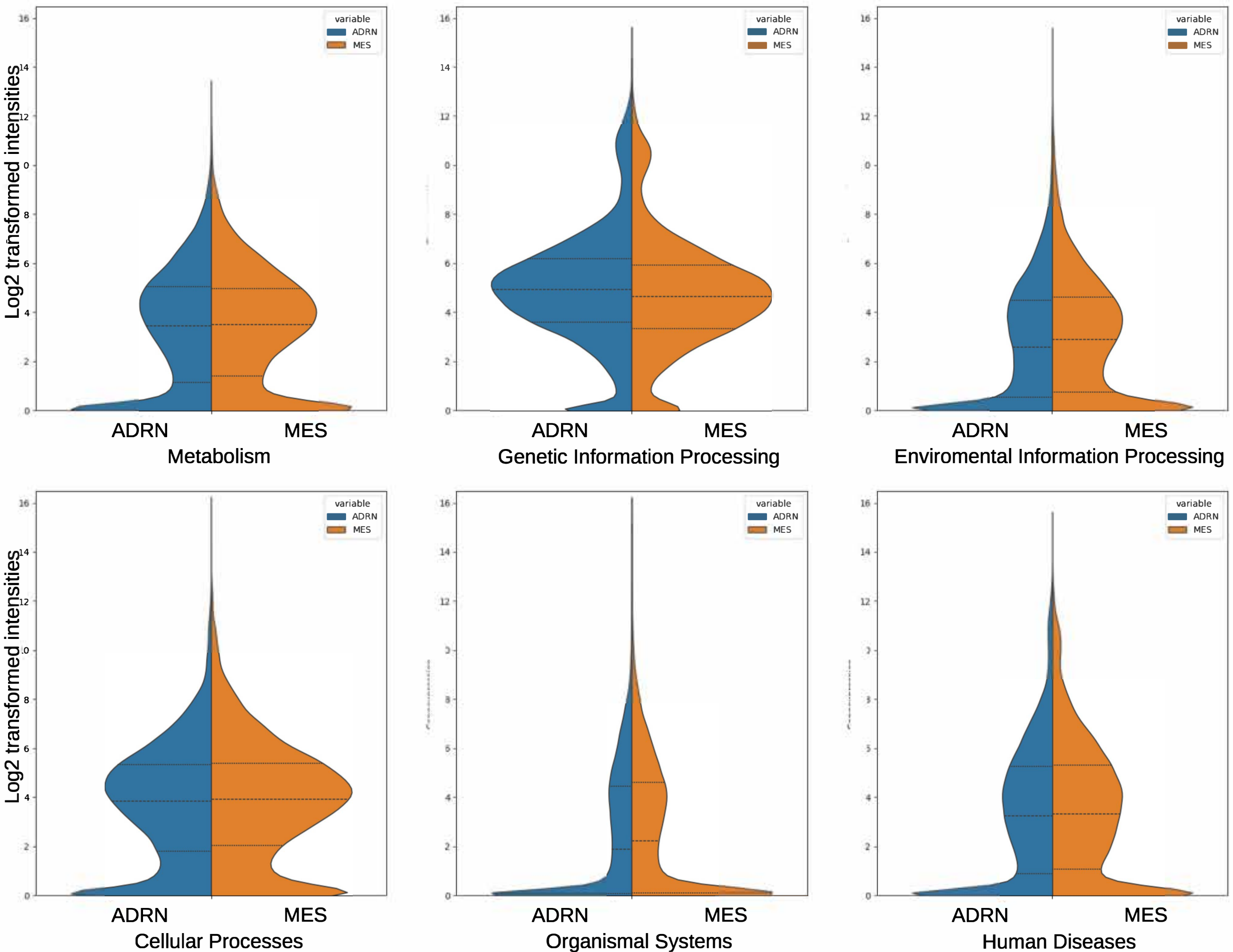

Sup. Fig. S10.Performance metrics from 10-fold cross-validation for Neuroblastoma sub-types

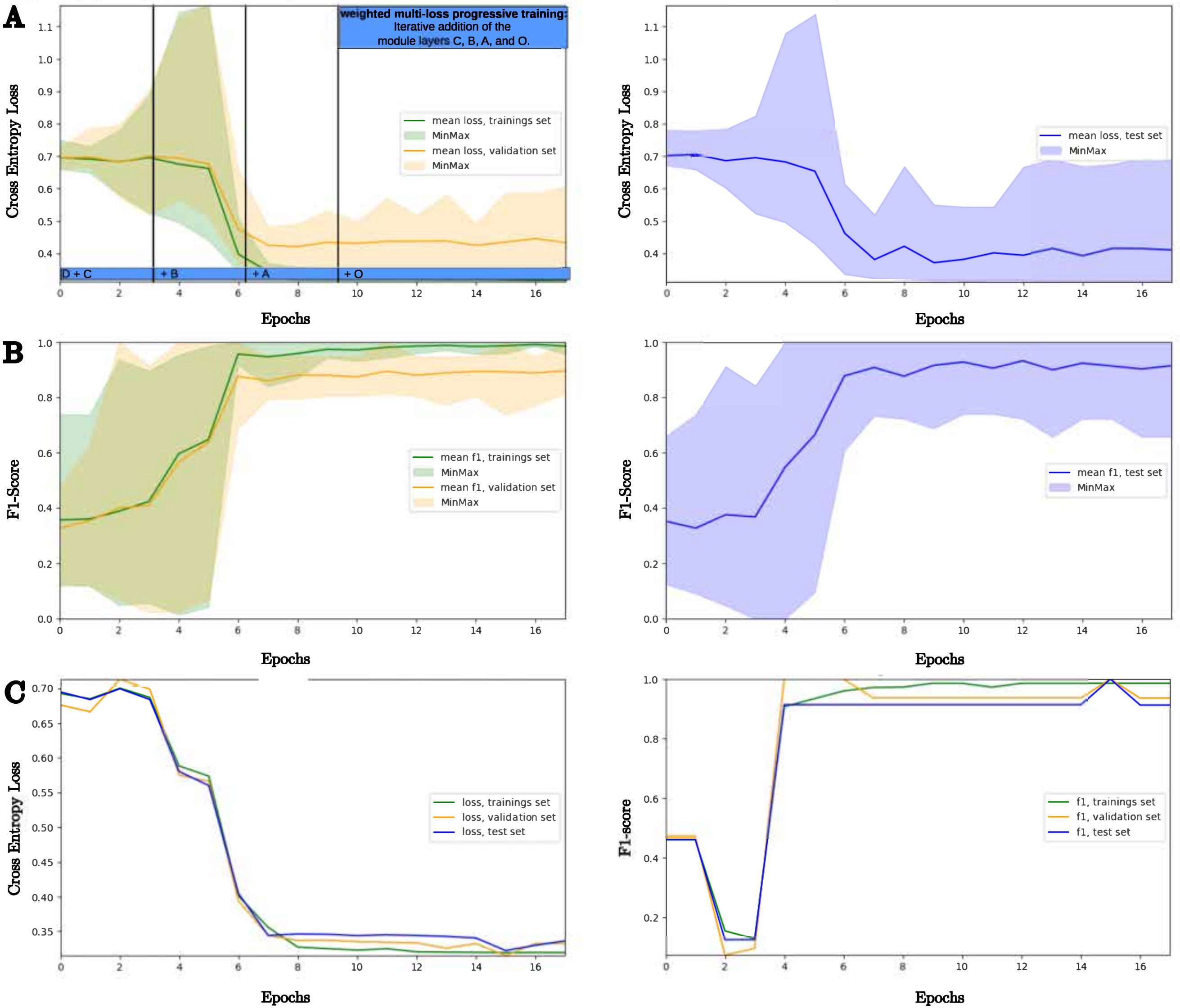

Supplement: Supplementary file 1 [file biomolecules-14-01501-s001.zip › Supplemental Figures S1-S10.pdf]
